# Supplementary material for: Are carbon emissions trading and green financial instruments synergistic? -Comprehensive quantitative research based on content analysis
Source: PLoS One. 2024 Mar 7;19(3):e0298601. doi: 10.1371/journal.pone.0298601 (PMC10919601; doi:10.1371/journal.pone.0298601)
Supplement: S1 Data — (ZIP) [file pone.0298601.s001.zip › Data/地级市Z_F工作报告.zip!/╕╩╦α/╟∞╤⌠╩╨/└┤╫╘╣1⁄2╓┌║┼í╛┬φ┐╦╩2╛▌═°í┐_2016.docx]

各位代表：

　　现在，我代表市人民政府，向大会作政府工作报告，请予审议，并请政协委员和列席人员提出意见。

　　“十二五”发展及2015年工作回顾

　　过去的五年，是我市经济社会实现大发展、大跨越的五年。五年来，在省委、省政府和市委的正确领导下，我们紧紧依靠和团结带领全市人民，在转变发展方式中化解经济下行压力，在聚焦精准扶贫中破解发展难题，在全面深化改革中激发内生动力，在坚持真抓实干中推动富民兴市，全面完成“十二五”规划各项约束性指标，基本实现预期性目标，经济社会发展取得了重大成就。

　　——坚持扩总量、稳增长，综合实力大幅提升。2015年，全市完成生产总值609.43亿元，是“十一五”末的1.86倍；规模以上工业增加值287.76亿元，是“十一五”末的2.1倍；地方固定资产投资1081.3亿元，是“十一五”末的2.6倍；社会消费品零售总额204亿元，是“十一五”末的2.2倍；财政大口径收入147.83亿元，是“十一五”末的2.5倍；一般公共预算收入55.4亿元，是“十一五”末的1.9倍；银行业金融机构存贷款余额分别达到814.7亿元、584亿元，是“十一五”末的2.3倍和4.2倍；城乡居民人均可支配收入分别达到23426元、6945元，是“十一五”末的1.9倍和2.2倍。

　　——坚持聚合力、出实招，扶贫攻坚成效显著。全面打响以通村公路为牵引的基础设施建设和以农民“五变”为内容的富民产业培育“两大扶贫攻坚战”。实施整村推进项目423个、整乡推进项目21个。新修通村公路6096.5公里，是2010年以前全市通村公路总里程的7倍，全市行政村通畅率提高到91.3%，实现了通乡油路全覆盖。建成农村供水工程3.3万处，解决了87万群众和学校师生安全饮水问题。完成农网改造5000多公里，改造低电压村（组）896个。改造农村危房（窑）9.72万户，实施易地扶贫搬迁4.5万人。全市2507个机关单位、3.29万干部，对口帮联537个贫困村、14.58万贫困户，累计落实帮扶物资30.45亿元，帮办实事15万件。全市贫困人口由2011年底的93.49万人减少到28.5万人左右，贫困面由40.55%下降到12.4%左右，贫困户人均可支配收入由1212元增加到3300元左右。

　　——坚持抓开发、促转化，能源产业持续壮大。石油开发快速推进，生产原油3186万吨，加工原油1588万吨，分别是“十一五”期间的2.1倍和3.2倍。煤炭开发初见成效，沙井子、甜水堡及修编后的宁正矿区总体规划获得国家发改委批复，刘园子煤矿建成投产，核桃峪煤矿“四井”贯通，正宁电厂获得国家发改委核准，环县电厂列入全省火电建设规划。天然气、煤层气、页岩气勘探开发稳步推进。环县南湫、毛井一期风电场相继建成投用，累计发电10.56亿度。庆阳经济技术开发区升级为国家级经济技术开发区的请示已经省政府同意上报国务院待批，17个工业集中区入驻企业298户，集聚效应和辐射带动功能进一步提升。

　　——坚持调结构、育特色，富民产业蓬勃发展。苹果产业引进推广“海升模式”和“西农技术”，栽植面积达到151.6万亩。草畜产业在“中盛模式”的带动下，建成各类规模养殖场1104个，发展规模养殖户8.3万户。全市肉牛、肉绒羊、生猪、肉鸡饲养量分别达到75万头、560万只、141万头、1590万只。瓜菜产业规模扩大，种植面积达到150万亩以上。粮食总产量累计达到768万吨，比“十一五”期间增加139万吨。新型农业产业化经营主体快速发展，兴办农产品加工企业256户，带动农户27.19万户；发展农民专业合作社5711个、家庭农场100个，特色产业收益占到农民收入的一半。

　　——坚持挖潜力、上规模，第三产业提速增效。电商进农村“百乡千村万店”工程深入实施，8县（区）电子商务服务中心投入运营，环县、宁县、华池、镇原进入国家和省上农村电子商务综合示范县行列。“互联网+”成为推动发展的新亮点，带动交通运输、餐饮住宿、健康养老、信息咨询等服务业提质增效。文化旅游融合发展迈出新步伐，建成了周祖农耕文化产业园、南梁红色旅游景区、天富亿生态民俗村等重点景区。

　　——坚持夯基础、破瓶颈，城乡面貌发生巨变。通过全方位争取，《陕甘宁革命老区振兴规划》《陇东能源基地开发规划》《千亿级循环经济产业链实施方案》分别得到国务院、国家能源局和省政府批复。庆阳机场4C级改扩建工程建成投用，架起了我市通往外界的“空中走廊”；青兰高速和福银高速庆阳段建成通车，西平铁路穿境而过，结束了我市不通高速、没有铁路的历史；建成二级公路4条，实现了县县通二级公路的目标。完成了扬黄人饮续建工程、环县苦咸水淡化等重点骨干水利项目，全市供水能力达到2.8亿立方米，有效灌溉面积达到53.9万亩。新建改建330千伏输变电工程3个、110千伏输变电工程8个、35千伏输变电工程15座。统筹推进市区、县城和小城镇建设，市区在全省率先建成地下综合管廊79公里，建成区面积扩展了近3倍；全市城镇建成区面积累计达到151平方公里，城镇化率由“十一五”末的23.79%提高到33.46%。深入实施“再造一个子午岭”和“固沟保塬”工程，累计造林359万亩，修建梯田146.39万亩，完成流域治理1832.4平方公里，全市森林覆盖率由“十一五”末的23.94%提高到

　　27.48%。

　　——坚持推改革、扩开放，发展活力竞相迸发。紧盯重点领域、关键环节，全面深化改革。进一步取消、调整和下放行政审批权限，市级行政审批事项由2010年的856项减少到182项，减幅达75%。农村“三权”改革稳步扩大，累计流转土地133.7万亩，流转率达到20%。探索推进资源配置市场化改革，组建了庆阳能化、交通投资、水务发展、庆阳城投、文化旅游投资5个集团公司，搭建了资源开发、地企融合的载体平台；市公共资源交易中心累计完成交易额294亿元，节约公共资金5.39亿元。深化财政收支管理改革，积极推进国库集中支付制度，实现了预算执行全程动态监控。成功引进甘肃银行、兰州银行、交通银行等商业银行在我市设立分支机构。推进市区公共交通管理体制改革，组建了庆阳公共交通集团，方便了市民出行。充分利用两个市场、两种资源，扩大对外开放。主动融入新丝绸之路经济带和西安经济圈，与意大利卡布拉罗拉市结为友好城市，与美国埃德蒙市达成结为友好城市意向，与印中工商会签署了经贸合作协议，在白俄罗斯、斯里兰卡等国家成功举办经贸文化合作项目推介会，在北京、上海等国内多个城市举办招商引资项目推介会和签约仪式。对外贸易进一步扩大，完成外贸进出口22.9亿元。

　　——坚持惠民生、增福祉，社会事业全面进步。教育事业稳步发展，全市义务教育普及率达到99.8%，人均受教育年限比“十一五”末增加了1.2年；2015年高考二本以上进线率达到30.8%，比“十一五”末提高了9.2个百分点；实施全面改薄项目1968个，新建公办幼儿园415所，各级各类学校基础条件明显改善。科技应用推广步伐加快，实施科技项目721项，推广新技术新品种700项，科技对经济增长的贡献率达到52%。医疗卫生条件显著改善，实施市、县、乡医院改扩建项目107个，建成标准化村卫生室1081个，市人民医院、市中医院成功晋升“三甲”。文化事业蓬勃发展，文化产业不断壮大，建成乡（镇）综合文化站57个、乡村舞台847个、文化集市48个、农家书屋31个。竞技体育水平不断提高，全民健身氛围日益浓厚，成功举办了第二届全国红运会、第十二届省运会和第八届省民运会。就业创业工作持续加强，招考安置高校毕业生18126人，非公企业对接安置4077人，城镇新增就业20.56万人，城镇登记失业率控制在3%以内。社会保障待遇水平稳定提高，城市、农村低保和特困人员保障标准分别由“十一五”末的2004元、950元、1600元提高到3720元、2434元、4114元，全民参保人数由55万人增加到206万人。社会福利和养老服务业快速发展。建成各类保障性住房1.57万套，发放租赁补贴1.3亿元。为民办实事累计投入资金235.5亿元，办理民生实事175件。民族乡村“1+9”发展规划深入实施，对口支援甘南藏区建设成效显著。社会治安综合治理、市场秩序整顿、安全生产专项整治扎实开展，食品药品、特种设备、产品质量安全监管持续加强，“六五”普法工作获全省优秀，全市社会大局和谐稳定。统计、气象、地震、档案、老龄、残疾人、红十字会和双拥、国防动员、民兵预备役等工作都取得了新的进步。

　　——坚持转作风、提效能，政府建设持续加强。自觉接受人大依法监督和政协民主监督，积极支持人大代表、政协委员开展视察调研，累计办理人大代表建议378件、政协委员提案1175件。扎实推进依法行政，决策水平进一步提升。严格落实中央“八项规定”、国务院“约法三章”、省委“双十条规定”和市委“实施细则”，深入开展政府系统党的群众路线教育实践活动、“三严三实”专题教育，审计监督、行政监察、政务公开力度加大，“四风”问题得到有效遏制。全市清退办公用房59217平方米，“三公”经费和会议经费支出逐年下降。

　　各位代表，刚刚过去的2015年，我们按照省委、省政府和市委的决策部署，积极应对经济下行压力加大、能源价格持续下跌的不利影响，着力稳增长、调结构、转方式，着力打基础、破瓶颈、增后劲，着力促改革、惠民生、保稳定，较好地完成了市三届人大五次会议确定的目标任务。

　　一是精准扶贫取得新成效。制定出台了“1+20”精准扶贫精准脱贫意见方案，探索形成了“五个一”扶贫工作新模式，打出了脱贫攻坚的“组合拳”。预计可实现西峰、正宁、合水3个县（区）整体脱贫，157个贫困村、19.35万贫困人口稳定脱贫。新修通村公路324条、3173公里，是我市农村公路史上投入资金最多、建设里程最长、群众受益面最大的一年。5个县（区）实现了村村通沥青（水泥）路，交通运输部在我市召开了全国农村公路现场会。新建维修各类农村供水工程9028处，新增安全饮水9.67万人。新建农电线路4793公里，改造低电压村（组）683个。新修梯田35.8万亩，新增流域治理面积433平方公里。建设易地扶贫搬迁安置点106处，搬迁贫困群众4801户、2.2万人。改造农村危房（窑）3.23万户，解决了10.6万群众的安居问题。

　　二是产业培育迈出新步伐。能源开发持续推进，生产原油776万吨，增长7.5%；加工原油257万吨。刘园子煤矿通过竣工验收，生产原煤38万吨；甜水堡2号煤矿投入试生产；正宁电厂完成主机招标。瑞华天然气综合利用项目完成主体工程及配套设施，永欣石化9万吨干气芳构化项目具备试生产条件。农业结构持续调整，推广全膜双垄沟播面积353万亩，粮食总产量达到163.1万吨，实现了连续八年丰产。新栽苹果10.2万亩、改造老果园3万亩。新建规模养殖场210个，发展规模养殖户1.1万户，肉牛、肉绒羊、生猪饲养量持续增长。种植瓜菜151.2万亩。完成苗林结合培育107万亩。第三产业持续壮大，建成乡级电商服务站81个，村级电商示范店509个，带动发展各类网店6153个，从业人员1.8万人，线上交易额突破15亿元。陇东汽车城、嘉鑫国际建材城和长庆桥陇禧物流园建设加快推进。庆阳民俗文化产业园、黄帝文化景区、陕甘红军纪念园等文化旅游项目进展良好，南梁红色旅游景区、天富亿生态民俗村被评定为国家4A级旅游景区。

　　三是基础建设实现新突破。纵贯我市南北、群众翘首企盼的银西高铁庆阳段开工建设，庆阳机场改扩建工程航站区全面建成，西合二级公路全线贯通。小盘河、五台山、莲花寺3个水库开工建设，马莲河水利枢纽工程进入国家“十三五”重大水利项目名录。市区庆化大道中段、东大街改造和市南区雨洪集蓄保塬生态项目建成投用。城市棚户区改造前期工作扎实推进，争取授信支持额度居全省前列。丽晶新天地、中元世贸等城市商务综合体项目建设进展顺利。

　　四是体制机制焕发新活力。行政审批制度改革步伐加快，“三张清单一张网”全面建立，市、县权责清单全部上线公布，取消、调整和下放行政审批事项215项。“三证合一、一照一码”商事登记制度全面实行，新注册各类企业3237户，新增各类市场主体2.2万户。农村综合改革稳步实施，3个县（区）、35个乡（镇）土地确权登记全面完成；新增流转土地25.3万亩；集体林权制度改革持续推进，国有林场试点改革任务全面完成。投融资改革成效明显，农村产业发展资金合作社实现了行政村全覆盖，发放自有资本贷款1.32亿元；与兰州银行合作，发放“金桥工程”贷款12.5亿元、省列精准扶贫贷款20.38亿元；与省农发行签订600亿元额度的中长期项目贷款协议；与省建行、甘肃银行合作启动了中小微企业“助保贷”业务；庆阳农村商业银行挂牌成立。公共服务领域改革持续深化，探索推进陇东报社、市广播电视台市场化改革和国有演艺公司股份制改革；公立医院综合改革全面推开，分级诊疗、双向转诊制度有序推进，实现基本药品零差率销售，患者住院费用和药品价格明显下降；机关事业单位养老保险制度、县以下机关单位职务与职级并行制度和公务用车改革有序推进。

　　五是社会事业有了新进步。职业教育体系趋于完善，庆阳职业技术学院正式挂牌招生；“美丽园丁”教育基金项目成功落户庆阳，北师大庆阳附属学校开工建设，掀开了我市借助优质教育资源和高端教育人才提升教育水平的新篇章；学前教育不断加强，中小学D级危房全面消除，薄弱学校改造计划如期完成。庆阳大剧院、传媒技术业务用房建设进展顺利，7个县城城市数字影院建成投用；深入开展“文化进万家”和“千台大戏送农村”活动，极大地丰富了群众的文化生活。市第二人民医院全面竣工，市妇女儿童医院开工建设，建成标准化村卫生室113个，医疗服务能力进一步提升。建成市、县、乡社会保障服务平台35个，培训城乡劳动力64万人次，输转劳务58.8万人。医疗救助和临时救助受益面进一步扩大，困难群众基本生活得到有效保障。大力实施“精准治污”行动，市区空气质量优良天数达到293天，主要河流水质稳定改善。

　　各位代表，过去的五年，是我市经济实力提升最快、社会事业发展最好、城乡面貌变化最大、人民群众得到实惠最多的时期。这些成绩的取得，是省委、省政府坚强领导、亲切关怀的结果，是市委统揽全局、科学决策的结果，是市人大、市政协支持监督、共同促进的结果，是全市广大干部群众团结拼搏、努力奋斗的结果。我谨代表市人民政府，向各位代表和政协委员，向全市各行各业的建设者，向各民主党派、工商联、无党派人士和人民团体，向离退休老同志，向驻庆人民解放军、武警官兵和中央、省属驻庆单位，向所有关心支持庆阳发展的社会各界人士，致以崇高的敬意，表示衷心的感谢！

　　各位代表，五年的发展实践和奋斗历程，使我们深深地体会到，推动庆阳经济社会发展，必须做到“五个坚持”：

　　一是必须坚持弘扬南梁精神。以“面向群众、坚守信念、顾全大局、求实开拓”为主要内容的南梁精神，是引领我市经济社会发展的思想航标和制胜法宝。我们必须继续大力弘扬优良革命传统，善于从人民群众中汲取奋进的智慧，注重凝聚各个方面的力量，朝着全面小康的宏伟目标阔步前行。

　　二是必须坚持发展第一要务。把发展作为破解一切矛盾和问题的根本出路，按照既定的工作思路，持续不懈地推进“三个生态”创建、打好“两大扶贫攻坚战”、建设“两个千亿级产业链”等重大战略部署，全力促进经济社会持续健康发展。

　　三是必须坚持为民利民导向。始终把人民群众放在心中最高位置，多谋民生之利，多解民生之忧，多办民生之事，着力解决人民群众行路、饮水、住房、入学、医疗、就业等切身利益问题，让改革发展成果更多更公平地惠及全体人民。

　　四是必须坚持扩大改革开放。用改革的办法、创新的举措、开放的胸襟破解发展难题，破解体制机制性障碍，进一步优化发展环境，提升行政效能，为创业创新注入动力，为加快发展增添后劲。

　　五是必须坚持抓落实干到位。“实”字当头、“干”字为先，扑下身子去做、去干，持之以恒抓执行、抓落实，推动各项工作取得实实在在的效果。

　　各位代表，在看到成绩的同时，我们也清醒地认识到，政府工作与人民群众的期望还有一定差距，全市经济社会发展中还存在着一些亟待解决的困难问题。一是贫困面大，凸显了全面小康的艰巨性。全市仍有28.5万左右的贫困人口，而且绝大多数集中在基础条件差的边远山区，贫困程度深，脱贫难度大，部分群众生活还比较困难，如期实现整体脱贫摘帽、全面建成小康社会，任务相当艰巨。二是产业单一，凸显了经济结构的不合理性。石油石化产业占比大，地方工业十分弱小，经济增长受市场波动影响明显；农业产业化程度不高，现代服务业水平低，发展中不平衡、不协调的问题比较突出。2015年，受原油价格持续低迷的影响，石化产业增产不增值，导致全市生产总值和财政收入未能完成年初计划目标。三是基础滞后，凸显了项目建设的紧迫性。交通道路、城乡供水和城市集中供热等基础设施不完善，项目谋划、论证和储备不足，部分在建项目施工进展慢，投资对经济增长的拉动作用发挥得还不够充分。四是落实乏力，凸显了作风建设的必要性。面对新常态、新任务和新要求，一些政府工作人员出现了怕出事不干事、怕担责不担当的问题，抓工作的方法不活，抓落实的力度不大，依法行政的能力有待提高。对此，我们要认真研究，采取有效措施，着力加以解决。

“十三五”规划目标及2016年工作任务

　　“十三五”时期，是深入落实“四个全面”战略布局、推动我市经济社会发展转型升级的关键阶段，也是全面建成小康社会的决胜阶段。以广域视角纵览宏观大势，我们面对的风险挑战复杂严峻。世界经济仍将延续疲弱复苏的态势，市场有效需求不足，大宗能源类商品价格持续跌落。国内经济下行压力仍在加大，部分行业产能严重过剩、资源约束趋紧，发展的不确定性、不稳定性因素增多。以战略眼光审视微观趋势，我们面临的利好机遇叠加呈现。全球以“互联网+”为代表的新一轮科技革命和产业变革，拓展了经济发展新空间；我国加快实施“一带一路”战略，出台的稳定经济增长、实施供给侧改革、降低实体经济成本等新举措，为我市发展带来了新契机。特别是国家和省委、省政府为革命老区“量身定做”的倾斜支持政策叠加效应正在陆续释放，一批重大项目开工建设，我市发展前景将会更加广阔。我们必须敏抓机遇、因势利导、主动作为，努力把庆阳改革发展事业不断推向前进。

　　综合考虑面临的形势和任务，今后五年全市经济社会发展的基本思路是：深入贯彻党的十八大和十八届三中、四中、五中全会及习近平总书记系列重要讲话精神，全面落实省委、省政府和市委的总体部署，坚持“四个全面”战略布局，坚持发展第一要务，紧扣“一个总体目标”（与全省全国一道全面建成小康社会），树立“五个发展理念”（创新、协调、绿色、开放、共享），创建“三个生态”（风清气正的政治生态、活力迸发的经济生态、山清水秀的自然生态），实施“四大战略”（产业兴市、工业强市、生态立市、依法治市），打造“五区一极”（革命老区精准脱贫攻坚示范区、国家能源战略重要接续区、西北绿色农产品加工贸易区、特色文化旅游融合发展创新区、黄土高原生态安全屏障试验区，全省区域发展的重要增长极），加快建设幸福美好新庆阳。

　　奋斗目标是：通过持续努力，2017年全市贫困面下降到4%以内，实现整体稳定脱贫；2020年全市生产总值跃上千亿元台阶，与全省全国一道全面建成小康社会。

　　战略重点：

　　一是围绕全面小康目标，着力打造革命老区精准脱贫攻坚示范区。把全面小康、脱贫攻坚作为全局工作的重中之重，以“六个精准”为主要抓手，深化完善“五个一”工作模式，全力抓好脱贫攻坚“一号工程”。持续打好富民产业培育攻坚战，发展壮大苹果、草畜、瓜菜、苗林四大产业，有效增加农民收入。持续打好基础设施建设攻坚战，着力解决农村群众出行、用水、用电、住房等方面的实际困难，实现村组通硬化路，基本消除城市棚户区、城中村，推进城乡公共服务均等化。重点推进庆阳机场4D级改造，加快发展通用航空；建成银西高铁庆阳段和甜水堡至罗儿沟圈高速公路等重大交通项目，实现县县通高速，着力构建快捷高效的立体交通网络体系；实施马莲河水利枢纽工程等重大水利项目，有效满足城乡群众生产生活用水需求。

　　二是围绕资源高效开发，着力打造国家能源战略重要接续区。持续加大原油产能建设，每年新增产能100万吨，加快实施庆阳石化600万吨炼油升级改造项目，谋划启动1000万吨升级改造工程，力争到2020年原油产量、加工量突破“双千万吨”。加快煤炭生产转化千亿级产业链建设，力争到2020年煤炭产能达到6000万吨以上，煤电装机达到1200万千瓦以上。加快天然气、煤层气、页岩气勘探开发，到2020年实现市区、县城管道输气全覆盖。积极发展风力发电、光伏发电等新能源产业，到2020年风电、光伏发电装机分别达到210万千瓦和40万千瓦。

　　三是围绕农业增产增效，着力打造西北绿色农产品加工贸易区。坚持宜农则农、宜牧则牧、宜果则果、宜林则林，加快推进农业结构调整。粮食种植面积稳定在700万亩左右、产量150万吨以上，保障粮食安全；苹果种植面积达到200万亩以上，肉牛、肉绒羊、生猪、肉鸡饲养量分别达到100万头、1200万只、200万头、1亿只，苗林结合培育总面积达到700万亩以上，瓜菜面积达到150万亩以上。支持壮大“农字号”龙头企业、农民专业合作社、家庭农场、种养大户等经营主体，健全基层农业服务体系，提高农业机械化水平。大力发展农产品精深加工业，引导农业走“产出高效、产品安全、集约经营”的道路。

　　四是围绕开放包容共享，着力打造特色文化旅游融合发展创新区。以华夏文明传承创新区和陇东南始祖文化历史区建设为抓手，把文化旅游产业作为新兴支柱产业，加快景区景点基础设施建设，积极融入西安、延安、银川经济文化旅游圈，打造旅游“金三角”，搭建文化+旅游、体育、岐黄中医药等产业发展平台，进一步扩大“红色南梁、岐黄故里、周祖圣地、民俗庆阳”四大文化品牌的知名度和影响力。

　　五是围绕生态文明建设，着力打造黄土高原生态安全屏障试验区。牢固树立“绿水青山就是金山银山”的理念，积极创建国家级生态市和省级园林城市。大力发展循环经济，促进形成绿色生产方式和消费方式。深入实施再造一个子午岭、固沟保塬、资源开发区治理“三大生态工程”，每年造林100万亩、流域治理600平方公里。到2020年，全市森林覆盖率达到35%、水土流失治理程度达到70%以上。

　　2016年，是实施“十三五”规划的开局之年，也是全面建成小康社会决胜阶段的开局之年。做好2016年的政府工作，要把中央和省上的部署与我市实际紧密结合起来，主动适应、把握和引领经济发展新常态，坚持稳中求进工作总基调，统筹稳增长、调结构、惠民生、防风险各项工作，确保经济提质增效、社会和谐稳定，努力实现“十三五”发展良好开局。

　　预期目标是：生产总值增长8.5%，规模以上工业增加值增长9%，固定资产投资增长10%，社会消费品零售总额增长9%，财政大口径收入和一般公共预算收入均增长8%，城镇居民人均可支配收入增长10%，农村居民人均可支配收入增长13%。各项约束性指标控制在省上下达的计划之内。

　　工作重点：

　　（一）精准施策、决战决胜，在扶贫开发上集中攻坚。

　　深入实施“五项脱贫基础工程”。农村公路建设工程，新修农村公路1724公里，实现村村通油路，打通制约贫困乡村发展和群众出行的“最后一公里”。农村安全饮水工程，新建、维修供水工程1.48万处，解决21.6万农村人口的安全饮水问题，农村饮水安全率达到88%。农网提质升级工程，新建（改造）配电线路1249公里；治理低电压村（组）284个，实现自然村（组）动力电全覆盖。易地扶贫搬迁和危房（窑）改造工程，新建安置点176处，搬迁建档立卡贫困群众5517户、3.48万人；实施危房（窑）改造26364户。梯田建设工程，新修梯田30万亩，全市梯田化率达到65%以上，力争正宁、合水整县实现梯田化。

　　统筹实施“三大行动”。增收产业培育行动，种植全膜粮食350万亩、小杂粮100万亩以上；新栽苹果10万亩，改造郁闭低效园6万亩；更新种植紫花苜蓿100万亩，新建规模养殖场200个，肉牛、肉绒羊、生猪、家禽饲养量进一步增加；种植瓜菜145万亩，其中籽用南瓜10万亩。继续实施“精准扶贫专项贷款”工程，大力推广政策性农业保险，对贫困户保险保费予以补助。特色品牌创建行动，加快“三品一标”农产品认证登记，大力宣传推介庆阳苹果、白瓜子、黄花菜、陇东黑山羊等绿色品牌，提升我市农特产品知名度。龙头企业引领行动，扶持贫困村建立专业合作组织，创新完善“龙头企业+合作社+家庭农场+基地+农户”的运营机制，培育市级示范性农民专业合作社、家庭农场100个。实施好环县6个行政村、200户光伏扶贫试点工程。

　　着力推进“三个落实”。落实帮联机制，建立“双联”和脱贫攻坚行动领导责任机制，完善以减贫结果为导向的考核评价体系，凝聚形成责任共担、同向攻坚的强大合力。落实培训计划，整合农牧、人社、扶贫、科技等培训资源，培训贫困乡村劳动力60万人次，为每个贫困家庭至少培养一个科技明白人，实现培训一技、发展一人、脱贫一家。落实脱贫任务，力争年内实现华池、庆城、宁县3县整体脱贫，166个贫困村、13万人稳定脱贫。

　　（二）链式延伸、多元互补，在工业发展上聚力突破。

　　深度开发油气资源。抓住国家适度加快常规油气勘探开发的政策机遇，积极支持油田企业扩能上产，力争原油产量达到815万吨、炼量完成350万吨；加强与中石油集团的对接，力争庆阳石化600万吨炼油升级改造项目启动实施。建成瑞华天然气综合利用、永欣石化干气芳构化项目，加快能化集团“气化庆阳”调峰项目建设。

　　稳步推进煤炭开发转化。促使甜水堡2号煤矿达产达标，加快核桃峪、新庄煤矿建设，力争马福川煤矿、正宁电厂、环县电厂和西峰热电联产项目开工建设。紧扣煤炭清洁利用和深加工，超前谋划煤炭转化项目，争取陇东特高压直流外送工程列入国家“十三五”电网建设规划予以实施。充分利用国家“推进煤矿气权矿权统一”的政策，督促入驻企业同步勘探开发煤层气，实现效益最大化。

　　积极开发风能和太阳能资源。抢抓国家支持非化石能源产业发展的机遇，加快毛井二期20万千瓦和芦家湾、紫坊2个5万千瓦风电项目建设，力争年内并网发电。做好环县小南沟、甜水堡二期、紫坊二期风电项目前期工作。实施好环县、镇原、华池、西峰4个县（区）太阳能光伏发电项目，建成光伏电站5处，容量7万千瓦。

　　壮大地方工业。把发展地方工业作为应对经济下行压力、调整优化产业结构的着力点和突破口，认真落实促进非公经济和中小微企业发展的各项政策，支持存量企业开展要素整合、资产重组和技术改造；围绕能源开发配套服务、特色农产品加工、文化旅游业和现代服务业等重点领域，新办地方工业，发展混合所有制经济。加大财政支持力度，扩大“助保贷”范围，引导金融机构增加贷款规模，帮助停产企业恢复生产，促使在产企业达产达标。引导支持非公经济主体特别是本土企业家转换经营观念，建立现代企业制度，由“家族制”向“股份制”转变，增强企业实力和抵御市场风险能力。支持推动大众创业、万众创新，扶持有一定潜力的个体工商户做大做强，培育一批“双创”示范企业。支持西峰制药、中盛农牧、中庆农产品等地方工业项目加快建设，确保能化集团装备制造园建成投产，提升园区承接产业转移能力，形成吸附优质生产要素的“磁场效应”。

　　（三）创新引领、促进融合，在第三产业上谋求提升。

　　创新发展现代服务业。深入实施“互联网+”行动计划，引导传统服务业升级改造，加快“线下”实体经营与“线上”网络经营融合发展、提升效益。支持信息咨询等新兴服务业加快发展，创新培育“文化+旅游+服务”业态，强化景区景点道路和配套服务设施建设，加大宣传推介力度，积极推动南梁红色旅游景区创建国家5A级旅游景区。

　　多元扩大居民消费。适应大众消费需求新变化，大力促进信息、住房、旅游、文化娱乐、健康养老等新兴消费，提供个性化、多样化消费产品，培植中低端、大众化消费热点。

　　积极完善市场体系。加快市区10大专业市场、5大物流园建设。持续推进宽带进村、“百乡千村万店”、新农村现代流通网络工程，健全完善商贸流通体系。加强市场监管，切实维护消费者的合法权益。

　　（四）上争外引、加快实施，在项目建设上高效推进。

　　抓好项目谋划争取。瞄准国家政策导向、投资和产业发展方向，做深做细项目谋划、论证、储备和规划编制工作，加强对上衔接争取，使我市更多项目纳入国家、省上规划“盘子”；用足用好国家支持中西部基础设施建设等政策机遇，争取更多中央资金投向我市重点领域和薄弱环节；深化政府与社会资本合作，争取更多项目进入国家、省上PPP项目库和重点推介项目名录。

　　加强基础设施建设。加快银西高铁庆阳段建设进度，推进庆阳至平凉、环县至褚家湾铁路项目前期工作；开工建设甜水堡至罗儿沟圈、打扮梁至庆城、南梁至太白、西峰至镇原杨涧河高速公路；完成309国道庆阳段和6条省道改建年度任务。加快小盘河、五台山、莲花寺水库建设，开工建设董志塬水厂，做好马莲河水利枢纽工程和扬黄工程甜水堡调蓄水库项目前期工作。开工建设宁县110千伏输变电工程，加快庆中、宁县330千伏和西峰温泉、庆城110千伏输变电工程前期工作。

　　扩大对外招商引资。坚持“走出去”与“引进来”相结合，一方面紧盯大企业、大项目实施“定向突破”，另一方面积极走出去举办重大项目和名优产品推介活动，力争更多有实力的国有企业和大型民营企业落户我市。积极推进光彩事业陇原行暨“民企陇上行”活动和“千企帮千村”行动，促使更多的招商引资项目落地实施，确保到位资金增长10%以上。

　　（五）科学规划、建管并重，在城市发展上彰显特色。

　　科学引领规划布局。坚持以人民为中心的城市发展思想，科学定位城镇发展方向，合理谋划城镇空间布局，充分彰显城镇历史文化积淀和黄土地域风情，积极推进“多规合一”，完善控制性详细规划和专项规划，着力解决“千城一面”的问题。严格规划的刚性约束，做到不符合规划的项目不审批，确保“一张蓝图”干到底。

　　统筹推进城镇建设。紧扣市区、县城、小城镇三大重点，推动地下与地上同步建设，老城区改造提升与新城区功能配套协调推进。突出抓好城市道路、供热供气、供排水、污水垃圾处理、生态绿地、休闲广场等公共配套设施建设。积极争取进入海绵城市、智慧城市和地下管廊城市试点行列。开工实施市区供水水质提升、管网改造工程，完成东区集中供热工程，完善整体服务功能。进一步扩大住房公积金覆盖范围，积极稳妥地推进房地产行业去库存，实施棚户区改造货币化补偿安置。

　　着力强化城市管理。加强城市网格化、精细化管理，实施治堵保畅工程，进一步理顺市区公交运营体制，有效解决交通拥堵、噪音污染和“脏乱差”等“城市病”，建设宜居城市。加强住宅小区物业管理，努力为市民营造舒心舒适的生活环境。加强市民教育，提升市民素质，积极创建全国文明城市。加快推进户籍制度改革，促进有能力在城镇就业生活的农业转移人口和其他常住人口市民化。

　　（六）绿色发展、协调推进，在生态建设上持续发力。

　　推进“再造一个子午岭”工程。加大苗林结合培育力度，扎实推进天保二期、三北五期、退耕还林还草、植树护路等工程，完成造林100万亩以上。落实封山禁牧措施，巩固造林绿化成果，拓宽苗木销售渠道，让群众依靠苗林产业发展增加收入。

　　推进“固沟保塬”工程。实施塬、峁、梁、沟、坡立体防御，加大沟头、沟坡、沟道治理，新建水保拦蓄工程1850座，新建淤地坝10座，完成水土流失治理面积600平方公里，有效遏制水土流失，控制塬面萎缩，构建生态防线。

　　推进“资源开发区生态治理”工程。建立地企共建共担的生态保护与修复责任机制，加大油区、矿区、水源地等重点区域综合治理，完成生态恢复年度任务；落实环境影响评价和环保“三同时”制度，扎实开展大气污染、水污染防治行动，严厉查处环境违法行为；加强国土资源管理，持续推进城乡环境综合整治，加快建设“美丽乡村”，让庆阳蓝天永驻、绿水长流。

　　（七）冲破藩篱、释放活力，在改革开放上加快步伐。

　　深化重点领域改革。行政审批改革方面，持续推进简政放权、放管结合、优化服务工作，完善“三张清单一张网”，落实“网上行权”；稳妥推进事业单位分类改革；持续深化商事制度改革。农村综合改革方面，加快推进土地确权登记颁证工作；放活土地经营权，积极推进农村集体经营性建设用地入市工作；持续深化集体林权制度配套改革。国资国企改革方面，进一步理顺“一区四园”、市属国有企业职责定位，加强国有资产管理，推动政企、政资、出资人职能与公共管理职能分开。财政金融改革方面，健全完善财政资金统筹使用机制，深化预算制度改革，有效防范和化解政府债务，着力提高精细化管理水平；创新金融产品，发展普惠金融，强化金融扶贫，推动农村产业资金合作社规范运行；加强对小额贷款、投资担保公司等机构的监督管理，规范各类融资行为，依法打击非法集资和信用违约行为，防范区域性、系统性金融风险；充分发挥保险杠杆作用，积极推出金融+保险创新产品。公共服务领域改革方面，深化公立医院改革，推进医药分开，实行分级诊疗；推进中医药综合改革试点，建立覆盖城乡的基本医疗卫生制度。深化文化体制改革，鼓励引导社会力量发展文化产业，培育地域文化产品和要素市场。完成市县公务用车制度改革。 　　持续扩大对外开放。积极融入丝绸之路经济带建设，加强与中亚、西亚及中东欧国家经贸往来，深化与西安经济圈、周边地区和长三角、京津冀等发达地区的经济合作，全面提升经济外向度。借助丝绸之路（敦煌）国际文化博览会、广交会等节会平台，发展会展经济，把我市的特色农畜产品、民俗文化产品推向省外域外市场，实现区域、企业、产业融合发展。

　　（八）以人为本、民生至上，在社会事业上统筹发展。

　　大力发展各项社会事业。优先发展教育，全面推进各级各类学校标准化建设，实施好“美丽园丁”基础教育质量提升协同创新计划、教育信息化工程和“乡村教师支持计划”，完成北师大庆阳附属学校主体工程。加快薄弱学校改造和有实际需求的行政村幼儿园建设，加强职业技术教育，推动教育均衡发展。加大科技推广应用力度，加快科技大市场建设，建立创新研发激励机制，发挥科技特派员和“三区”科技人才作用，提高科技成果推广应用水平。推进城乡文化建设，加快庆阳民俗文化产业园、传媒技术业务用房、大剧院等重点工程建设，做好图书馆等项目前期工作，加强文化遗产传承保护，促进文化繁荣发展。深入推进全民健身活动，办好全市第十三届运动会。提升医疗卫生服务能力，启动市人民医院二期工程前期工作，加快市妇女儿童医院和县乡医疗卫生机构改扩建，建设村级标准化卫生室56个，实施乡（镇）卫生院“温暖工程”25个；强化医疗卫生专业人才队伍建设，提升医疗服务水平；加强计划生育工作，全面落实二孩生育政策。加强就业创业，深入实施“高校毕业生就业促进计划”和“大学生创业引领计划”，做好复转军人、困难家庭就业帮扶工作，促进创业带动就业。有组织地推动劳务输转，新增城镇就业5万人，力争城镇登记失业率控制在4%以内。强化社会保障，实施全民参保计划，提高城乡低保标准，积极发展社会福利和残疾人、慈善事业，关爱农村留守儿童、妇女，健全城乡养老服务体系，创新社会救助机制，筑牢保障困难群众基本生活的防线。加强民族宗教工作，促进少数民族乡村加快发展。

　　扎实办好民生实事。在认真落实中央和省上惠民政策、完成省上确定的为民办实事项目的基础上，市财政投入资金3.36亿元，全力办好11件民生实事：（1）将城乡居民基础养老金补助标准每人每月提高10元；（2）落实贫困村扶贫互助资金市级配套资金；（3）解决7万贫困人口饮水困难问题；（4）实施市区东区、南区供水管网建设工程；（5）建设市区公共自行车服务体系；

　　（6）与北师大合作培训学科紧缺教师；（7）实施千名医护人员素质提升培训计划；（8）继续实行婚前免费医学检查；（9）完成市区10条街巷综合治理工程；（10）新建城乡社区老年人日间照料中心200个；（11）完成279个行政村“乡村舞台”建设。

　　全力维护和谐稳定。深化平安庆阳建设，重视抓好生产安全和公共安全，进一步强化道路交通、油气管道、校园、危险化学品、消防、煤矿和非煤矿山、特种设备、食品药品、环境等重点领域监管，保障人民群众生命财产安全。强化“七五”普法宣传教育，引导群众自觉尊法学法守法用法，自觉践行社会主义核心价值观。着力打造责任信访、阳光信访、法治信访，深入推进领导干部下访接访，把矛盾消除在基层源头。创新社会治理，健全完善立体化社会治安防控体系和应急处置机制，加强各类风险防控，提高防灾减灾和应对突发事件的能力。依法严厉打击盗窃、赌博、买卖婚姻和暴力犯罪等违法行为，确保社会和谐稳定。

加强政府自身建设

　　全面完成今年和“十三五”各项任务，对政府自身建设提出了新的更高的要求。我们必须自觉践行“三严三实”，认真履行职责，对人民负责，受人民监督，让人民满意。

　　（一）创新理念增活力，着力建设服务政府。以战略思维谋划推动发展，加强对重大问题的战略研究和顶层设计，科学指导推进政府工作。始终把人民的期盼作为奋斗目标，关心群众疾苦，倾听群众心声，了解群众意愿，把更多的公共资源向民生倾斜，更好地为基层、为企业、为群众服务，着力优化干事创业、投资发展环境，真心实意为群众办实事、做好事、解难事，努力提升群众的满意度。

　　（二）依法行政促规范，着力建设法治政府。严格按法定权限和程序履行职责，切实做到法定职责必须为、法无授权不可为。严格执行重大行政决策制度，启动实施地方立法工作，把政府活动全面纳入法治化轨道。强化政务公开，注重公开实效。自觉接受人大依法监督和政协民主监督，认真办理人大代表议案、建议和政协委员提案。加强诚信体系建设，提高政府的公信力。

　　（三）担当实干抓执行，着力建设高效政府。以钉钉子的精神抓落实，坚持说了就办、定了就干，一项一项破解难题，一件一件兴办实事，在实干中化解矛盾，在实干中加快发展。坚决治庸、治懒、治散，下实手解决不作为、慢作为、乱作为的问题，强化督促检查，确保各项工作高标准推进、高质量完成，切实增强政府的执行力。

　　（四）从严治政强约束，着力建设廉洁政府。严格落实中央“八项规定”、国务院“约法三章”和省、市各项规定。认真履行“一岗双责”，全面落实党风廉政建设主体责任体系，从严治政，廉洁从政。始终把纪律和规矩挺在前面，坚持用制度管人、管权、管事。严格审计监督、行政监察，加强工程建设、土地出让、公共资源交易等重点领域专项治理，严肃查处“四风”和腐败问题，确保干部清正、政府清明。

　　各位代表，“十三五”发展的征程已经开启，新起点昭示新希望，新蓝图呼唤新作为。让我们在市委的正确领导下，凝聚全民之力，激发拼搏之志，高扬奋进之帆，为全面建成小康社会、建设幸福美好新庆阳作出新的更大贡献！
